# Nuclear Magnetic Resonance-Based Metabolomic Analysis of the Anticancer Effect of Metformin Treatment on Cholangiocarcinoma Cells

**DOI:** 10.3389/fonc.2020.570516

**Published:** 2020-11-30

**Authors:** Jin Zhang, Caihua Hang, Ting Jiang, Shenghui Yi, Wei Shao, Wengang Li, Donghai Lin

**Affiliations:** ^1^ Department of Hepatobiliary Surgery, Xiang’an Hospital of Xiamen University, School of Medicine, Xiamen University, Xiamen, China; ^2^ College of Chemistry and Chemical Engineering, Key Laboratory for Chemical Biology of Fujian Province, MOE Key Laboratory of Spectrochemical Analysis & Instrumentation, Xiamen University, Xiamen, China; ^3^ Department of Physical Education, Xiamen University of Technology, Xiamen, China; ^4^ Affiliated Cardiovascular Hospital of Xiamen University, Medical College of Xiamen University, Xiamen, China

**Keywords:** cholangiocarcinoma, metformin, metabolomic, nuclear magnetic resonance (NMR), anticancer

## Abstract

Metformin is a widely prescribed anti-diabetes drug with potential utilities for cancer therapies. Several studies have related metformin to the reduced risk of cholangiocarcinoma (CCA), highlighting its potentialities for the treatments of CCA. However, the underlying molecular mechanisms remain elusive. Here, we demonstrated that metformin treatment could inhibit proliferations of the human CCA cell lines Mz-ChA-1 and QBC939 in dose-dependent manners. The NMR-based metabonomic analyses showed distinct discriminations between the metformin-treated (Met) and control (Ctrl) groups of both CCA cells. Characteristic metabolites were identified by a combination of multivariate statistical analysis of 1D ^1^H-NMR spectral data and the pair-wise t-test of metabolite levels. We then identified four significantly altered metabolic pathways based on the characteristic metabolites, including glucose metabolism, oxidative stress-related metabolism, energy metabolism, and amino acids metabolism. Comparing CCA cells with normal human umbilical vein endothelial cells (HUVECs), we found that metformin treatment profoundly promoted glycolysis and specifically increased the levels of BCAAs and UDP-GlcNAc, implying the occurrence of autophagy and cell cycle arrest in metformin-treated CAA cells. This work provides a mechanistic understanding of the anticancer effect of metformin treatment on CAA cells, and is beneficial to further developments of metformin as an anticancer drug.

## Introduction

### Background

Cholangiocarcinoma (CCA) is the second most common hepatic and biliary malignancy (1). Based on its anatomical location, CCA can be classified into intrahepatic, extrahepatic and distal CCA (1, 2). Although CCA is considered as an uncommon tumor and only accounts for 3% of all gastrointestinal tumors, the overall incidence rate of CCA has remarkably climbed in last several decades (3, 4). Surgical intervention offers the highest chance to cure for all types of CCA. Unfortunately, individuals with CCA are usually asymptomatic, and most of patients diagnosed with CCA can no longer benefit from surgical resection (4), leading to poor outcomes. Even when surgery is an option for selected patients, the 5-year survival rates are still very low. Both systemic chemotherapy and targeted radiation therapy have been also applied for the treatments of CCA. Nevertheless, these approaches usually fail to efficiently improve the prognosis of CCA (3). Thus, new therapy strategies are urgently needed for CCA.

As the most widely used first-line drug for the treatment of type 2 diabetes, metformin (N, N-dimethyl biguanide) has recently gained interests of investigators for its anticancer potentials. According to recent epidemiological data, cancer risk in diabetic patients taking metformin is significantly reduced relative to patients with other antidiabetic treatments (5–8). Moreover, numerous studies have reported that metformin has anticancer effects both *in vivo* and *in vitro* on various human cancers including CCA (9–13). These evidences indicate that metformin might have great potentials for CCA prevention and therapy.

As reported recently, metformin treatment can reduce the levels of mitochondrial metabolites, activate multiple mitochondrial metabolic pathways, and increase 18-FDG flux in breast tumors (14). Moreover, metformin can inhibit mitochondrial complex I and disrupt oxidative phosphorylation, thus resulting in alterations in the electron transport chain (ETC) (15, 16). The inhibition of complex I also causes energetic stress, then enhancing the activity of AMP-activated protein kinase (AMPK) (17, 18). Furthermore, metformin can activate AMPK through the lysosomal pathway, and the anticancer effect of metformin treatment might be not mere a consequence of disrupting metabolic processes such as ATP synthesis through oxidation phosphorylation (19). In addition, previous works also indicate that metformin exerts anticancer capacities by inhibiting the mammalian target of rapamycin (mTOR) through AMPK-dependent and AMPK-independent mechanisms (20, 21). However, molecular mechanisms underlying the anticancer effect of metformin treatment on CCA remain to be detailedly clarified.

Metabolomic analysis has been extensively applied to clarify molecular mechanisms of anticancer drugs (22–24). As metabolites are the final downstream products of gene transcription and translation, variations in metabolite levels reflect systemic changes of biological states. Several complicated signaling pathways could simultaneously bring out alterations in a metabolic pathway. Therefore, a comprehensive metabolomic analysis is of great significance for elucidating the molecular mechanisms of metformin for the treatments of CCA.

As two most efficient detection techniques, mass spectrometry (MS) and nuclear magnetic resonance (NMR) spectroscopy are frequently used in metabolomic analyses. Despite NMR spectroscopy shows lower sensitivities than MS, it offers many unique advantages (25). NMR possesses the capacity of readily identifying and conveniently quantifying absolute levels of compounds present in biological samples, including the compounds which are difficult either to be ionized or are required for derivatization to conduct MS detections. In addition, NMR is a method of choice for the identification of compounds with identical masses, including those with different isotopomer distributions (25, 26). Thus, we took advantage of NMR to perform metabolomic analyses.

In the present work, we demonstrated that metformin treatment profoundly suppressed proliferations of two human CCA cell lines (MZ-CHA-1 and QBC939) in dose-dependent manners. By performing NMR-based metabonomic analyses on cellular extracts, we indicated that metformin treatment induced marked variations in metabolic profiles and remarkable changes in metabolite levels as well as significant alterations in metabolic pathways for both CCA cells. Moreover, certain metabolite levels exhibited different changing trends between the CCA cells and normal human umbilical vein endothelial cells (HUVECs) after metformin treatment. These results shed new light on the molecular mechanisms of the anticancer effect of metformin treatment on CCA cells.

## Materials and Methods

### Cell Lines and Culture

Two typical human CCA cell lines MZ-CHA-1 and QBC939 were used in this work. MZ-CHA-1 was properly conserved in our laboratory, and QBC939 was obtained from Third Military Medical University. Both CCA cells were cultured in RPMI 1640 supplemented with 10% fetal bovine serum (FBS, Gemini, USA), 100 U/ml penicillin, and 100 U/ml streptomycin. Known as normal epithelial cells, primary HUVEC cells were isolated from the umbilical cord of a neonate as described previously (27), cultured in endothelial cell medium (ECM; ScienCell, USA), supplemented with 5% FBS, 1% endothelial cell growth supplement (ECGS; ScienCell, USA), 100 U/ml penicillin, and 100 U/ml streptomycin. All the cells were cultured in a 5% CO_2_ humidified environment at 37°C.

### Cell Viability Assay

The cell viability assay was performed on both CCA cells using a CellTiter 96^®^ AQueous One Solution Cell Proliferation Assay Kit (Promega, USA) according to the recommendations of the manufacturer. The cells were seeded in 96-well plates (5 × 10^3^ per well). After 12 h, the medium was replaced with test medium containing various concentrations of metformin (0.05, 0.5, 2, 5 mM), and the cells were incubated for a further 48 h. Then, 20 μl of MTS solution was added to each well. After 4 h incubation in dark, the absorbance of formazan was measured at a wavelength of 490 nm on a microplate reader (BioTek, USA). Statistical results were presented as the mean ± SEM. To compare cell viabilities between the four metformin-treated (Met) groups and the control (Ctrl) group of the CCA cells, we analyzed the data with ONE-WAY ANOVA followed by multiple post-hoc comparisons (Bonferonni/Tukeys) using the GraphPad Prism program (Version 6, GraphPad Software, USA).

### Colony Formation Assay

Both CCA Cells were planted into 6-well plates at a density of 1,000 cells per well and treated with test medium containing various concentrations of metformin for 7–14 days. Colonies were fixed with 4% paraformaldehyde for 20 min, and stained with 0.5% crystal violet in 20% ethanol for 30 min. The plates were washed with water for 3 times and photographed with camera. The Image J software (Version 1.52a, National Institutes of Health, USA) was used to calculate grey value in each well. The colony formation rates were calculated and analyzed by ONE-WAY ANOVA using GraphPad Prism.

### Sample Preparation

Both CCA cells were seeded in 10 cm diameter culture dishes at a density of 1×10^6^ per dish and treated with or without 0.5 mM metformin. After 48 h of incubation, the cells reached 80–90% of confluence, and the difference in the number of cells between the Met group and Ctrl group was less than 10%. Before harvest, medium was removed and cells were quickly washed by ice-cold PBS for 3 times. Vacuum suction was used to remove any residual liquid. Then, 3.0 ml of cold methanol was immediately added into the culture dish, and the cells were scraped, collected and transferred into a centrifuge tube. Thereafter, 3.0 ml of cold chloroform and 2.5 ml of water were subsequently added to the tube, and the mixture was fully vortexed. After 30 min of laying aside, samples were centrifuged at 12,000 g for 15 min at 4°C to separate two phase extracts. The aqueous phase was condensed with nitrogen stream and lyophilized by a vacuum freezing dryer.

The lyophilized aqueous metabolite extracts were dissolved in 550 μl of NMR buffer containing 50 mM K_2_HPO_4_/NaH_2_PO_4_ (pH 7.4), 0.05 mM sodium 3-(trimethylsilyl)-propionate-2,2,3,3-d4 (TSP), 10% D_2_O, and 0.02% NaN_3_. D_2_O was used for a field-frequency lock, and TSP provided the chemical shift reference. All the samples were vortexed, centrifuged at 12,000 g for 15 min at 4°C to remove any insoluble components. Supernatants were transferred to 5 mm NMR tubes for further analysis. Four groups of the CCA cells were used in the following NMR measurements including metformin-treated MZ-CHA-1 cells (Met-Mz) and QBC939 cells (Met-939) and untreated controls (Ctrl-Mz, Ctrl-939), together with metformin-treated HUVEC cells and untreated controls (Met-Hv, Ctrl-Hv).

### Nuclear Magnetic Resonance Measurements and Data Processing

NMR experiments were conducted on a Bruker Avance III 850MHz spectrometer equipped with a TCI cryoprobe (Bruker BioSpin, Rheinstetten, Germany) at 298 K. One dimensional (1D) ^1^H NOESY spectra were acquired using the pulse sequence (RD-90°-t1-90°-τ_m_-90°-ACQ) with water suppression during the relaxation delay and mixing time (19). RD was the relaxation delay (4 s), t1 was a short delay (4μs), and τ_m_ was the mixing time (10 ms). The spectral width was 20 ppm with an acquisition time per scan of 1.88 s (ACQ), and a total of 128 transients were collected into 64 K data points for each spectrum. The free induction delay (FID) signal was processed by a window function with a line broadening of 0.3 Hz, followed by Fourier transformation to obtain 1D ^1^H spectra. To assist in resonance assignments of metabolites, two-dimensional (2D) NMR spectra were recorded including ^1^H-^13^C heteronuclear single quantum coherence (HSQC) and ^1^H-^1^H total correlation spectroscopy (TOCSY) spectra.

The TopSpin 3.5 software (Bruker Biospin, Germany) was used to perform phase and baseline corrections of *the* NMR spectra. Chemical shifts were referenced to the CH_3_ resonance of TSP at 0 ppm. Peak alignments were further manually carried out with MestReNova Version 9.0 (Mestrelab Research S.L., Espain). Spectral regions of δ 9.40-(-0.5) were binned by 0.001 ppm and integrals of the segments were calculated. Regions of residual water resonances at δ 5.2–4.6 were removed to eliminate the distorted baselines from imperfect water saturation.

Integrals were normalized by the integral area of TSP to make the data directly comparable between the NMR spectra. Then, probabilistic quotient normalization (PQN) was performed to compensate for dilution-independent effects in MATLAB (Version 2011b, Math Works, USA).

### Resonance Assignments of Cellular Metabolites

Resonance assignments of aqueous metabolites were conducted using a combination of Chenomx NMR Suite (Version 8.1, Chenomx Inc., Edmonton, Canada) and Human Metabolome Data Base (HMDB) (http://www.hmdb.ca/) as well as relevant published references. For each cluster of a given metabolite, we compared the shape of the Chenomx preview spectral line (preview line) and the experimental 1D ^1^H spectral line (Exp line). Under the condition that the preview line was substantially contributing to the Exp line in the displayed region, and also the Exp line was similar to the standard 1D ^1^H spectral lines provided by Chenomx NMR Suite, the metabolite was assigned to be the compound in the Chenomx Compound Table.

The resonance assignments were further confirmed based on 2D ^1^H-^1^H TOCSY and ^1^H-^13^C HSQC spectra together with the standard ^1^H-^1^H TOCSY and ^1^H-^13^C HSQC spectra provided by Human Metabolome Data Base. For metabolites with highly overlapping peaks, non-overlapping peaks were selected to accurately calculate their spectral integrals. GraphPad Prism was used to calculate the averages and standard errors of metabolite levels.

### Multivariate Statistical Analysis and Identification of Significant Metabolites

The normalized spectral data were scaled by Pareto scaling and objected to the SIMCA-P 14.0 software (Umetrics, Sweden) for multivariate statistical analysis. An unsupervised approach, principal component analysis (PCA) was performed to reveal the trends, highlight outliers, and show clusters among the samples. A supervised approach, partial least-squares discriminant analysis (PLS-DA) was subsequently conducted to improve the classification between the groups of samples. Cross-validation was performed with a random permutation test (999 cycles) to evaluate the robustness of the PLS-DA model. The model is considered credible if all the Q2-values on the left are lower than the original point at the right, and the regression line of the Q2-points intersects the vertical axis below zero.

Two criteria derived from the PLS-DA loading plot were used to identify significant metabolites primarily responsible for the metabolic discrimination: variable importance in the projection (VIP), and the correlation coefficient (r) of the variable relative to the first predictive component (tp1). The loading plot was reconstituted in MATLAB. The critical value of the correlation coefficient (r) was defined based on the degree of freedom (df), which were determined as n1+n2-2 with n1 and n2 as the respective number of samples of the two groups in the PLS-DA model. Variables with VIP >1 and |r|> the critical value of p = 0.01 were marked by red color; variables with VIP >1 and |r| between the critical values of p = 0.05 and p = 0.01 were marked by orange; variables with VIP ≤1 or |r|< the critical value of p = 0.05 were marked by blue. Variables colored in red and orange were related to significant metabolites.

Furthermore, hierarchical cluster analysis (HCA) with Pearson distance measure and Ward clustering algorithm was performed on the normalized NMR data to further confirm the metabolic clusters, using the module of Statistical Analysis provided by the MetaboAnalyst webserver 4.0 (http://www.metaboanalyst.ca/). In the HCA approach, each sample acted as a separate cluster initially and the algorithm proceeded to combine them until all samples belong to one cluster.

### Univariate Statistical Analysis and Identification of Differential Metabolites

Pair-wise student’s t-test was applied to quantitatively compare relative levels of metabolites between the Met and Ctrl groups. Metabolites with *p <*0.05 were identified to be differential metabolites. Characteristic metabolites were determined by a combination of the identified differential metabolites and the significant metabolites described above.

### Metabolic Pathway Analysis and Identification of Significant Pathways

Metabolic pathways were analyzed based on the characteristic metabolites using the module of Metabolites Set Enrichment Analysis (MSEA) provided by MetaboAnalyst 4.0. MSEA is extensively used to identify and interpret patterns of metabolite level changes in a meaningful context (28), which contains 88 metabolite sets functionally related to metabolic pathways. The statistical *p* value was calculated to evaluate the significance of the metabolic pathway. The metabolic pathway containing at least three enriched metabolites with *p <*0.05 was identified to be the significantly altered pathway (abbreviated as significant pathways).

## Results

### Metformin Inhibited Proliferations of Both CCA Cells in Dose-Dependent Manners

**Figure 1:**
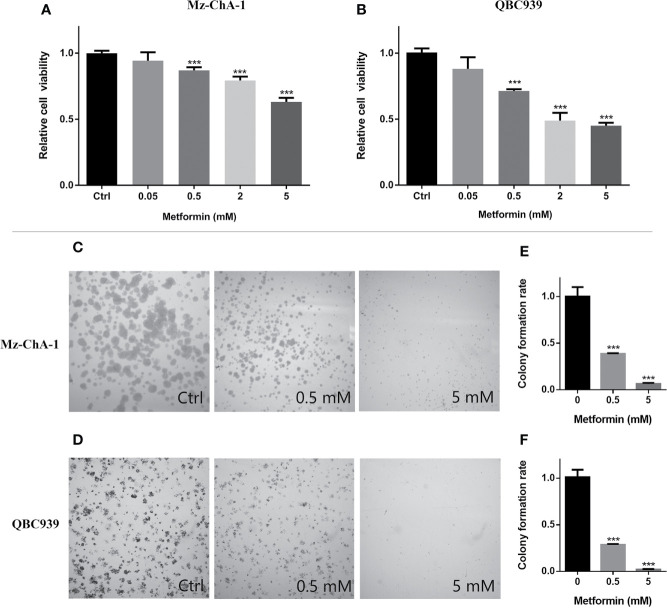
Metformin inhibited proliferations of both human cholangiocarcinoma (CCA) cells in dose-dependent manners. **(A, B)** Relative viabilities of the MZ-ChA-1 cells **(A)** and QBC939 cells **(B)** after 48 h incubation with various concentrations of metformin. *n =* 10, ***p < 0.001 relative to control cells. **(C, D)** Colony formations of MZ-ChA-1 cells **(C)** and QBC939 cells **(D)** before and after metformin treatment. **(E, F)** Quantification of colony formation rates of MZ-ChA-1 cells **(E)** and QBC939 cells **(F)**. *n =* 3, ***p < 0.001 relative to control cells.

To address the anticancer effect of metformin treatment on CCA, we conducted MTS assays and colony formation assays on both human CCA cells (MZ-CHA-1 and QBC939). The CCA cells were treated with various concentrations of metformin for both assays. MTS assays showed that metformin reduced cell viabilities in dose-dependent manners (**Figures 1A, B**). Moreover, relative cell viabilities were significantly decreased to 80–90% when the two CCA cells were treated with 0.5 mM metformin for 48 h. Such a treatment allowed the difference in the cell number between the Met and Ctrl groups was less than 10%. Our preliminary experiments showed that harvesting the two CCA cells at 48h provided maximum metabolite concentrations with smaller experimental errors. In colony formation assays, additionally, metformin at either 0.5 or 5 mM profoundly decreased the dimensions of colonies (**Figures 1C–F**). Thus, we treated the two CCA cells with metformin for 48 h in the following NMR-based metabonomic analyses.

### Metformin Markedly Changed Metabolic Profiles of Both CCA Cells

**Figure 2:**
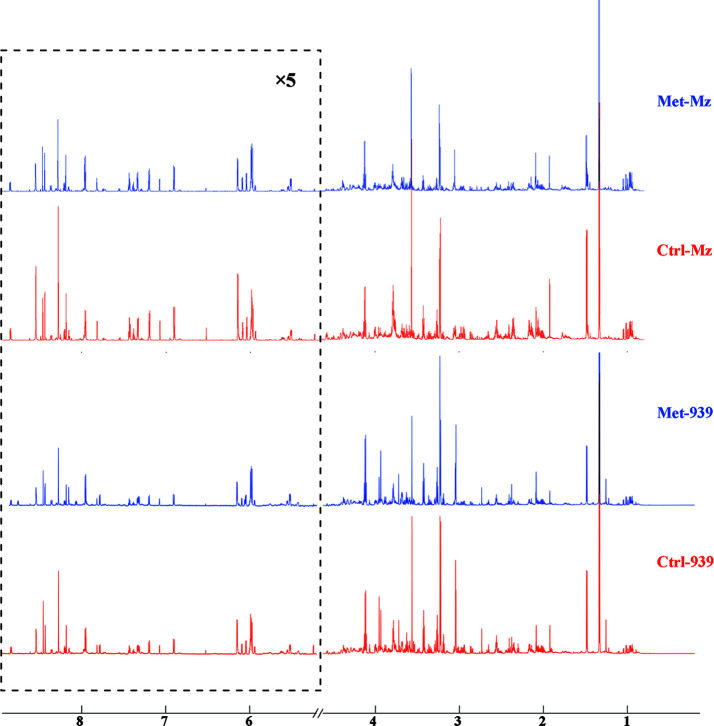
Averaged 1D ^1^H NOESY spectra of aqueous metabolites derived from the metformin-treated and control groups of both CCA cells. Spectral regions of 1.0–4.6 and 5.2–9.0 ppm were reserved, and the water region (4.6–5.2 ppm) was removed. Spectral regions of 5.2–9.0 ppm were scaled for 5 times. Met-Mz and Met-939 represent the groups of metformin-treated Mz-ChA-1 cells and QBC939 cells with metformin treatment, respectively. Ctrl-Mz and Ctrl-939 stand for the groups of Mz-ChA-1 cells and QBC939 cells without metformin treatment, respectively.

To reveal metabolic distinctions between the Met and Ctrl groups, we performed NMR-based metabonomic analyses on aqueous extracts derived from both CCA cells. The typical 1D ^1^H NOEYS spectra are shown in **Figure 2**. Resonance assignments of cellular metabolites were conducted (**Table 1**), and further confirmed by 2D ^1^H-^13^C HSQC and 2D ^1^H-^1^H TOCSY spectra (**Figure S1**). Totally, 41 metabolites were identified based on the NMR spectra.

**Table 1:** Resonance assignments of metabolites identified from 1D ^1^H-NMR spectra of aqueous extracts derived from the Met and Ctrl groups of both CCA cells.

Metabolite	δ ^1^H (ppm) and multiplicity	Moieties
2-Oxoglutarate	2.41(t), 3.00(t)	^γ^CH_2_, ^β^CH_2_
GABA	1.90(m), 2.29(t), 3.01(t)	^β^CH_2_, ^α^CH_2_, γCH_2_
Acetate	1.91(s)	CH_3_
Alanine	1.47(d), 3.78(q)	^β^CH_3_, ^α^CH
Asparagine	2.85(dd), 2.94(dd), 4.01(dd)	^β^CH_2_, ^β^CH_2_, ^α^CH
Aspartate	2.68(dd), 2.81(dd), 3.90(dd)	^β^CH_2_, ^α^CH
β-Alanine	2.54(t), 3.18(t)	^α^CH_2_, ^β^CH_2_
Choline	3.21(s), 3.51(dd), 4.04(t)	N(CH_3_)_3_, NCH_2_, CH_2_OH
Creatine	3.04(s), 3.93(s)	N-CH_3_, ^α^CH_2_
Creatine-P	3.05(s); 4.05(s)	N-CH_3_, CH_2_
Formate	8.46(s)	COH
Fumarate	6.51(s)	CH
Glucose	3.23(m), 3.40(m), 3.47(m), 3.53(dd), 3.83(m), 3.89(dd),	β(^2^H, ^3^H, ^5^H), α(^2^H, ^3^H, ^6^H)
Glutamate	2.08(m), 2.12(m), 2.34(m), 2.37(m), 3.75(m)	^β^CH_2_, ^β^CH_2_, ^γ^CH_2_, ^γ^CH_2_, ^α^CH
Glutamine	2.13(m), 2.45(m), 3.77(t)	^γ^CH_2_, ^β^CH_2_, ^α^CH
Glutathione	2.15(m), 2.54(m), 2.97(dd), 3.78(m), 4.20(q)	^β^CH_2_, ^γ^CH_2_, CHSH, ^α^CH&CH_2_NH, CHNH
Glycerol	3.55(m), 3.64(m), 3.78(tt)	^1.3^CH_2_OH, ^1,3^CH_2_OH, ^2^CH_2_OH
Glycine	3.57(s)	^α^CH_2_
GTP	4.24(m), 4.36(m), 4.58(t), 5.92(d), 8.13(s)	^5’^CH_2_, ^4’^CH, ^3’^CH, ^1’^CH, ^8^CH
Histidine	3.16(dd), 3.23(dd), 3.98(dd), 7.09(d), 7.0(d)	^β^CH_2_, ^β^CH_2_, ^α^CH, ^5^CH, ^2^CH
Hydroxyproline	2.14(ddd), 2.42(m), 3.36(ddd), 3.46(dd), 4.33(d), 4.35(d)	^β^CH_2_, ^β^CH_2_, ^β^CH_2_, ^δ^CH_2_, ^γ^CH, CH
Isoleucine	0.94(t), 1.01(d), 1.21(m), 1.42(m), 2.00(m), 3.67(d)	^δ^CH_3_, ^γ^CH_3_, ^γ^CH_2_, ^γ^CH_2_, ^β^CH, ^α^CH
Lactate	1.33(d), 4.11(q)	^β^CH_3_, ^α^CH
Leucine	0.96(d), 0.97(d), 1.69(m), 1.70(m), 1.73(m), 3.73(m)	^α^CH_3_, ^α^CH_3_, ^γ^CH, ^β^CH_2_, ^α^CH
Lysine	1.43(m), 1.49(m), 1.70(m), 1.91(m), 3.02(t), 3.75(t)	^γ^CH_2_, ^γ^CH_2_, ^δ^CH_2_, ^β^CH_2_, ϵCH_2_, ^α^CH
Methionine	2.10(m), 2.13(s), 2.17(m), 2.66(t), 3.78(m)	^β^CH_2_, SCH_3_, ^β^CH_2_, ^γ^CH_2_, ^α^CH
Myo-inositol	3.27(t), 3.52(dd), 3.61(t), 4.05(t)	^2^CH, ^4,6^CH, ^1,3^CH, ^5^CH
NAD	6.03(d), 6.08(s), 8.16(s), 8.20(m), 8.41(s), 8.82(d), 9.13(d), 9.32(s)	NH_2_, NH_2_(CO), ^δ^CH, ^β^CH, ^2^CH, ^γ^CH, ^α^CH,
PC	3.22(s), 3.60(t), 4.18(m)	N(CH_3_)_3_, NCH_2_, CH_2_OH
Ornithine	1.73(m), 1.83(m), 1.93(m), 3.05(t), 3.77(t)	^γ^CH_2_, ^γ^CH_2_, ^β^CH_2_, ^δ^CH_2_, ^α^CH
Phenylalanine	3.12(dd), 3.30(dd), 3.99(dd), 7.33(d), 7.37(t), 7.43(t)	^α^CH, ^β^CH_2_, ^β^CH_2_, ^α^CH, ^β^CH, ^γ^CH
Proline	1.99(m), 2.06(m), 2.34(m), 3.33(m), 3.41(m), 4.12(dd)	^γ^CH_2_, ^γ^CH_2_, ^β^CH_2_, ^β^CH_2_, ^δ^CH_2_, ^δ^CH_2_, ^α^CH
Putrescine	1.76(m), 3.05(m)	^β^CH_2_, ^α^CH_2_
Pyroglutamate	2.02(m), 2.39(m), 2.50(m), 4.17(dd)	^β^CH_2_, ^γ^CH_2_, ^β^CH_2_, ^α^CH
Pyruvate	2.36(s)	CH_3_
Serine	3.84(m), 3.94(dd), 3.98(dd)	^α^CH, ^β^CH_2_, ^β^CH_2_
GPC	3.23(s), 3.60(dd), 3.68(dd), 3.87(m), 3.94(m), 4.33(m)	N(CH_3_)_3_, ^1^CH_2_, ^2^CH_2_, ^1^CH_2_, ^3^CH_2_, ^3^CH_2_, ^1^CH_2_
Taurine	3.27(t), 3.43(t)	^1^CH_2_, ^2^CH_2_
Tyrosine	3.05(dd); 3.19(dd); 6.88(d), 7.18(d)	CH_2_, ^β^CH_2_,^β^CH, ^α^CH
UDP-GlcNAc	2.05(s), 3.55(t), 4.37(m), 7.92(d), 8.33(d)	CH_3_, ^4’’^CH, ^3’^CH, ^6^CH, ^2’’^NH
Valine	6.88(d), 7.18(d)	^γ^CH_3_, ^γ^CH_3_, ^β^CH, ^α^CH

Multiplicity: s, singlet; d, double; t, triplet; q, quartet; m, multiple; dd, double of double.

**Figure 3:**
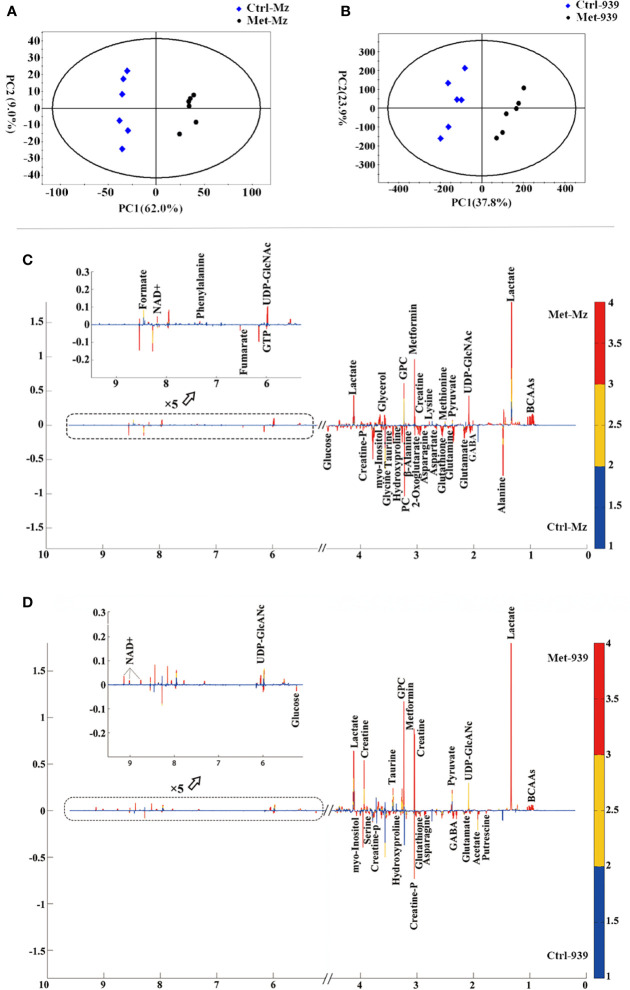
Multivariate statistical analysis of nuclear magnetic resonance (NMR) spectral data derived from aqueous extracts of the Met and Ctrl groups of both CCA cells. **(A, B)** PCA scores plots of the Met-Mz vs. Ctrl-Mz groups **(A)**, and the Met-939 vs. Ctrl-939 groups **(B)**; **(C, D)** Partial least-squares discriminant analysis (PLS-DA) loading plots for identifying significant metabolites primarily contributing to metabolic discriminations of the Met-Mz group from Ctrl-Mz groups **(C)**, and the Met-939 group from. the Ctrl-939 group **(D)**.

Both PCA and PLS-DA of the normalized NMR data were performed to evaluate the effects of metformin treatment on metabolic profiles of the CCA cells. All the samples are situated in the Hotelling’s T2 oval of the 95% confidence intervals. In the scores plot of either the PCA model or the PLS-DA model, each point represents a sample, and the distance between points reflects the degree of metabolic distinction. The PCA scores plots show distinct separations of the Met-Mz and Met-939 groups from Ctrl-Mz and Ctrl-939 groups, respectively, suggesting that metformin treatment markedly changed the metabolic profiles of both CCA cells (**Figures 3A, B**). The PLS-DA scores plots display improved metabolic separations of the Met-Mz and Met-939 groups from the Ctrl-Mz and Ctrl-939 groups (**Figures S2A, B**). The cross-validation plots indicate the high reliabilities of the PLS-DA models for both CCA cells (**Figures S2C, D**). Additionally, we conducted the HCA analyses to confirm the validities of the PCA and PLS-DA models (**Figures S2E, F**). The dendrogram plot of HCA illustrates that the Met-Mz group forms a separate cluster, while the Ctrl-Mz group belongs to another cluster (**Figure S2E**). The QBC939 cells displayed the similar HCA plot (**Figure S2F**). This result well supported those from PCA and PLS-DA.

### Metformin Profoundly Changed Metabolite Levels in Both CCA Cells

In the loading plots of the PLS-DA models (**Figures 3C, D**), variables colored in red, yellow, and blue are very significant, significant, and insignificant respectively, the upward or downward direction indicates the variable was upregulated or downregulated in the Met group compared with that in the Ctrl group. Expectedly, the levels of metformin were significantly increased in the Met-Mz and Met-939 groups relative to Ctrl-Mz and Ctrl-939, respectively, indicating that extracellular metformin was transported into both CCA cells. Significant metabolites were identified which were primarily responsible for metabolic separations of the Met-Mz and Met-939 groups from Ctrl-Mz and Ctrl-939 groups, respectively. After metformin treatment, 14 metabolites were increased and 18 metabolites were decreased in Mz-ChA-1 cells, while 10 metabolites were increased and 11 metabolites were decreased in QBC939 cells.

Relative levels of metabolites were represented by relative integrals measured from 1D ^1^H NOESY spectra. Quantitative comparisons of relative levels of metabolites between the Met-Mz and Ctrl-Mz groups, and between the Met-939 and Ctrl-939 groups were performed by using univariate statistical analysis (pair-wise student’s t-test), as shown in **Table 2**. Metabolites with statistical significances (*p* < 0.05) were identified to be differential metabolites. Totally, we identified 33 and 30 differential metabolites in Mz-ChA-1 and QBC939 cells, respectively.

**Table 2:** Quantitative comparisons of metabolite levels between the Met and Ctrl groups of both CCA cells based on relative integrals calculated from the 1D ^1^H-nuclear magnetic resonance (NMR) spectra.

Metabolite	Mz-ChA-1				QBC939			
	Mean ± SD		Significance	*p* value	Mean ± SD		Significance	*p* value
	Ctrl	Met			Ctrl	Met		
2-oxoglutarate	1.000 ± 0.114	0.590 ± 0.052	***	<0.001	1.000 ± 0.165	0.695 ± 0.073	**	0.002
GABA	1.000 ± 0.034	0.841 ± 0.085	**	0.002	1.000 ± 0.103	0.716 ± 0.036	***	<0.001
acetate	1.000 ± 0.262	0.804 ± 0.186	NS	0.166	1.000 ± 0.258	0.709 ± 0.151	*	0.038
alanine	1.000 ± 0.073	0.716 ± 0.044	***	<0.001	1.000 ± 0.115	0.958 ± 0.053	NS	0.438
asparagine	1.000 ± 0.076	0.749 ± 0.051	***	<0.001	1.000 ± 0.053	0.654 ± 0.028	***	<0.001
aspartate	1.000 ± 0.133	0.677 ± 0.090	***	0.001	1.000 ± 0.070	0.781 ± 0.156	*	0.011
β-alanine	1.000 ± 0.097	0.517 ± 0.041	***	<0.001	1.000 ± 0.111	0.797 ± 0.071	**	0.004
choline	1.000 ± 0.072	1.192 ± 0.084	**	0.002	1.000 ± 0.096	1.137 ± 0.108	*	0.043
creatine	1.000 ± 0.120	0.752 ± 0.065	**	0.001	1.000 ± 0.099	1.639 ± 0.105	***	<0.001
creatine-P	1.000 ± 0.042	0.729 ± 0.186	**	0.006	1.000 ± 0.135	0.692 ± 0.076	***	0.001
formate	1.000 ± 0.173	1.429 ± 0.357	*	0.024	1.000 ± 0.260	0.850 ± 0.260	NS	0.340
fumarate	1.000 ± 0.034	0.333 ± 0.070	***	<0.001	1.000 ± 0.348	0.484 ± 0.131	**	0.007
glucose	1.000 ± 0.426	0.473 ± 0.210	*	0.022	1.000 ± 0.321	0.210 ± 0.232	***	0.001
glutamate	1.000 ± 0.049	0.669 ± 0.043	***	<0.001	1.000 ± 0.119	0.650 ± 0.036	***	<0.001
glutamine	1.000 ± 0.066	0.694 ± 0.219	**	0.008	NS	NS	NS	NS
glutathione	1.000 ± 0.251	0.459 ± 0.158	**	0.001	1.000 ± 0.158	0.746 ± 0.104	**	0.008
glycerol	1.000 ± 0.063	1.504 ± 0.106	***	<0.001	1.000 ± 0.156	1.220 ± 0.333	NS	0.172
glycine	1.000 ± 0.053	0.847 ± 0.047	***	0	1.000 ± 0.072	0.937 ± 0.060	NS	0.129
GTP	1.000 ± 0.056	0.869 ± 0.035	***	0.001	1.000 ± 0.165	0.830 ± 0.054	*	0.037
histidine	1.000 ± 0.051	0.925 ± 0.084	NS	0.092	1.000 ± 0.291	0.988 ± 0.069	NS	0.921
hydroxyproline	1.000 ± 0.062	0.603 ± 0.071	***	<0.001	1.000 ± 0.098	0.609 ± 0.038	***	<0.001
isoleucine	1.000 ± 0.052	1.342 ± 0.088	***	<0.001	1.000 ± 0.100	1.345 ± 0.078	***	<0.001
lactate	1.000 ± 0.088	1.325 ± 0.069	***	<0.001	1.000 ± 0.101	1.512 ± 0.139	***	<0.001
leucine	1.000 ± 0.061	1.382 ± 0.082	***	<0.001	1.000 ± 0.100	1.361 ± 0.073	***	<0.001
lysine	1.000 ± 0.067	1.209 ± 0.023	***	<0.001	1.000 ± 0.120	0.610 ± 0.041	***	<0.001
methionine	1.000 ± 0.038	1.339 ± 0.070	***	<0.001	1.000 ± 0.041	1.005 ± 0.047	NS	0.856
myo-inositol	1.000 ± 0.038	0.663 ± 0.037	***	<0.001	1.000 ± 0.081	0.726 ± 0.042	***	<0.001
NAD+	1.000 ± 0.043	1.091 ± 0.051	**	0.008	1.000 ± 0.127	1.319 ± 0.124	**	0.001
PC	1.000 ± 0.189	0.609 ± 0.115	**	0.002	1.000 ± 0.290	0.886 ± 0.042	NS	0.363
ornithine	1.000 ± 0.071	0.974 ± 0.052	NS	0.478	1.000 ± 0.158	0.790 ± 0.067	*	0.014
phenylalanine	1.000 ± 0.047	1.193 ± 0.074	***	0	1.000 ± 0.059	0.940 ± 0.195	NS	0.486
proline	1.000 ± 0.089	0.911 ± 0.045	NS	0.055	1.000 ± 0.142	0.992 ± 0.079	NS	0.904
putrescine	1.000 ± 0.137	1.119 ± 0.109	NS	0.128	1.000 ± 0.159	0.530 ± 0.042	***	<0.001
pyroglutamate	NS	NS	NS	NS	1.000 ± 0.057	1.088 ± 0.039	*	0.011
pyruvate	1.000 ± 0.115	1.349 ± 0.278	*	0.017	1.000 ± 0.211	1.503 ± 0.324	**	0.010
serine	1.000 ± 0.092	1.023 ± 0.049	NS	0.597	1.000 ± 0.166	0.610 ± 0.052	***	0.000
GPC	1.000 ± 0.053	1.264 ± 0.020	***	<0.001	1.000 ± 0.123	1.454 ± 0.097	***	<0.001
taurine	1.000 ± 0.036	0.609 ± 0.053	***	<0.001	1.000 ± 0.091	1.273 ± 0.084	***	0.000
tyrosine	1.000 ± 0.046	1.037 ± 0.081	NS	0.354	1.000 ± 0.065	0.991 ± 0.071	NS	0.831
UDP-GlcNAc	1.000 ± 0.039	1.731 ± 0.190	***	<0.001	1.000 ± 0.430	1.648 ± 0.187	**	0.007
valine	1.000 ± 0.076	1.486 ± 0.085	***	<0.001	1.000 ± 0.116	1.371 ± 0.073	***	<0.001

The quantitative comparisons were conducted by using student’s t-test. Symbols ***, **, *, NS indicate differences between the Met and Ctrl groups were highly significant (p < 0.001), very significant (p < 0.01), significant (p < 0.05), insignificant (p ≥ 0.05), respectively. Red or blue color denotes that the metabolite level was elevated or reduced in the metformin-treated cells relative to control cells. NA, not applicable.

### Metformin Affected Similar Metabolic Pathways in Both Cholangiocarcinoma Cells

**Figure 4:**
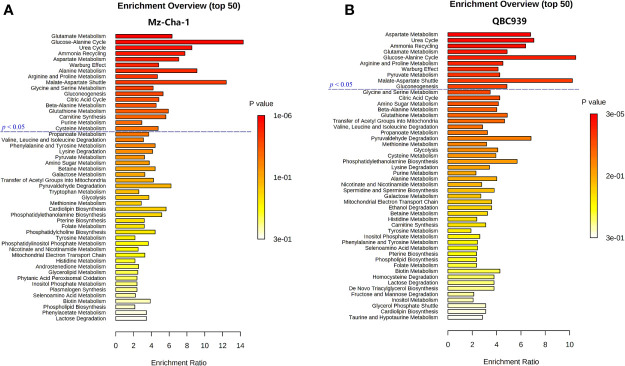
Metabolic pathways altered by metformin treatment in both CCA cells. **(A, B)** Top 50 metabolic pathways derived from metabolite sets enrichment analyses based on characteristic metabolites identified in MZ-ChA-1 cells **(A)** and QBC939 cells **(B)**.

**Figure 5:**
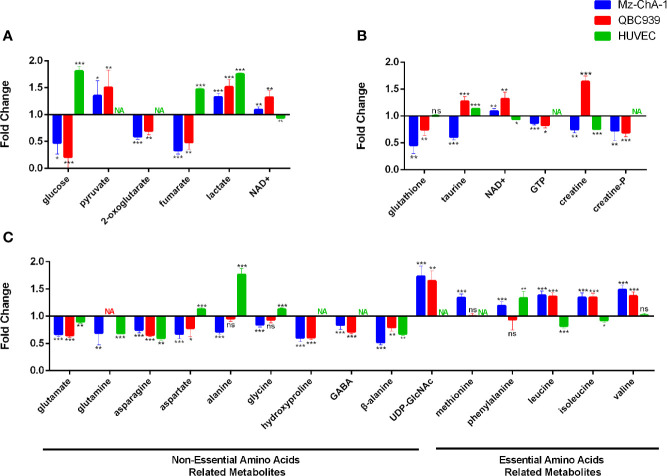
Metabolite levels in metformin-treated MZ-ChA-1, QBC939 and human umbilical vein endothelial cells (HUVEC) cells relative to their untreated controls. **(A–C)** Metabolites involved in glucose metabolism **(A)**, energy metabolism and oxidative stress-related metabolism **(B)**, and amino acids metabolism **(C)**.

Combining the identified differential metabolites with the significant metabolites described above, we obtained 32 and 29 characteristic metabolites in the Met-Mz and Met-939 cells compared with their controls, respectively. The characteristic metabolites were then subjected to metabolic pathway enrichment analysis. Top 50 enriched pathways are shown in **Figure 4**. The ranking of enriched metabolic pathways in the Met-Mz cells was highly similar with the Met-939 cells, and the pathways with higher significances were closely related to four major metabolic pathway clusters, including glucose metabolism, oxidative stress-related metabolism, energy metabolism, and amino acids metabolism. Metformin-induced level changes of characteristic metabolites involved in the four major metabolic pathway clusters were quantified for Met-Mz vs. Ctrl-Mz, and Met-939 vs. Ctrl-939 (**Figure 5**). Most metabolite levels exhibited consistent changing trends in the two CCA cells. The metformin-treated CCA cells showed upregulated levels of pyruvate, lactate and NAD^+^, and downregulated levels of glucose, GTP and TCA cycle intermediates (2-oxoglutarte; fumarate). Furthermore, most of the detected non-essential amino acids were decreased, and some of the detected essential amino acids (methionine, phenylalanine, valine, leucine, and isoleucine) tended to be increased in the metformin-treated CCA cells. These results suggest that metformin treatment has similar effects on the two CCA cell lines.

### Metformin Differently Affected the Levels of Certain Metabolites in Cholangiocarcinoma Cells

**Figure 6:**
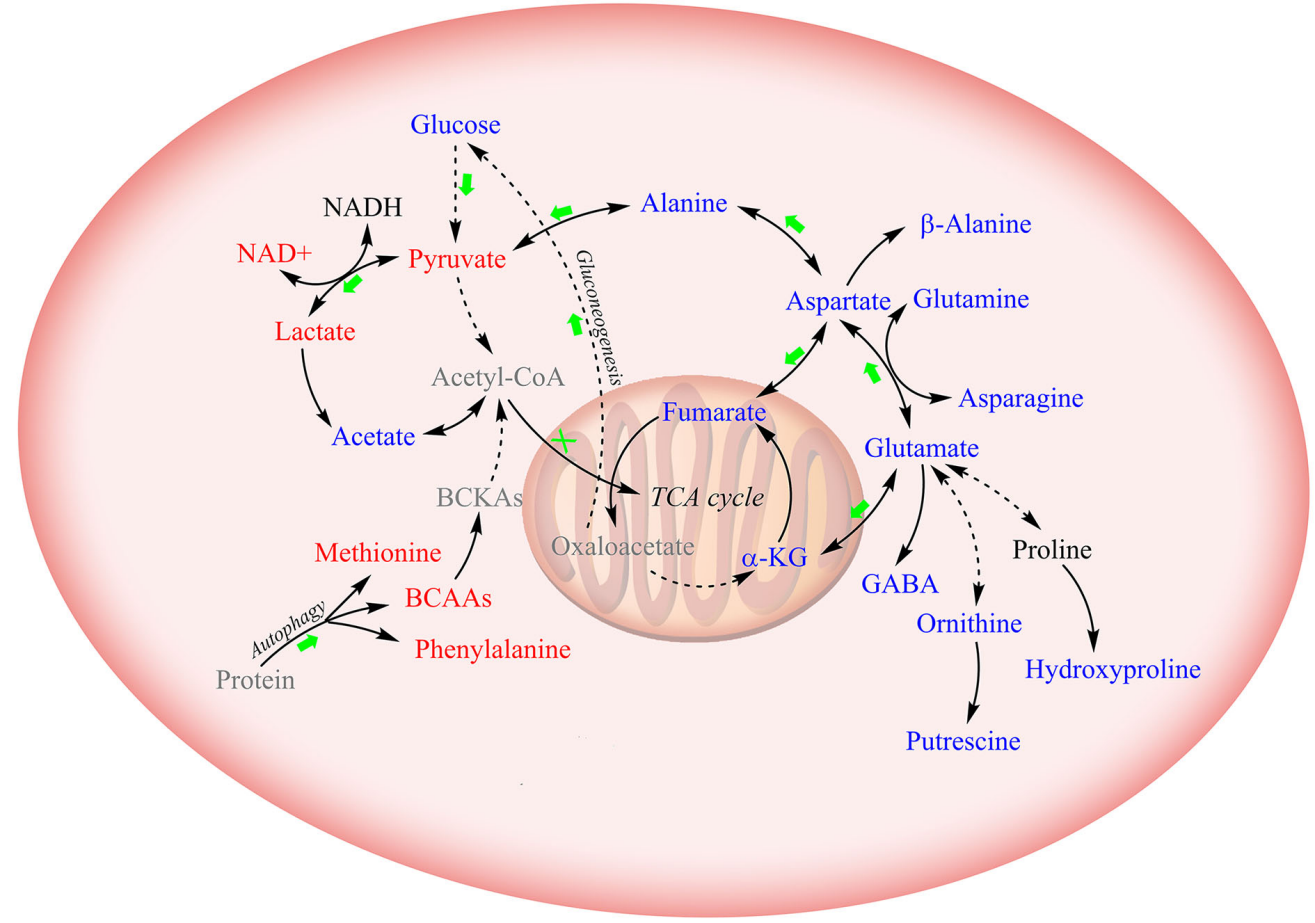
Schematic diagram for the effects of metformin treatment on metabolic pathways in CCA cells. Solid lines: direct conversion; dotted line: indirect conversion. Metabolites colored in red, blue, and black represent increased, decreased, and unchanged by metformin treatment in at least one CCA cell lines (MZ-ChA-1 and/or QBC939 cells), respectively. Metabolites displayed in grey were not detected by 1D ^1^H-NMR spectra. Green arrows and red crosses denote potentially promoted and inhibited metabolic pathways in metformin-treated CCA cells relative to their control counterparts, respectively.

To assert the unique effects of metformin treatment on CCA cells, we further assessed metformin-induced level alterations of metabolites involved in the four major metabolic pathway clusters in normal HUVEC cells (**Figure 5**). The HUVEC cells shared several metabolite levels with the two CCA cells, including the increased levels of lactate, decreased levels of glutamate, glutamine, asparagine, and β-alanine. After metformin treatment, glucose, fumarate, and alanine were remarkably decreased in CCA cells but increased in HUVEC cells, while NAD^+^, UDP-GlcNAc, and BCAAs were increased in CCA cells but either decreased or unchanged in HUVEC cells. To visualize the effects of metformin treatment on the metabolic pathways of the CCA cells, we projected the characteristic metabolites onto a metabolic map (**Figure 6**). Both the primarily changed characteristic metabolites and significantly altered metabolic pathways provide new insights into molecular mechanisms underlying the anticancer effect of metformin treatment on CCA cells.

## Discussion

As reported previously, metformin is transported into hepatocytes through the organic cation transporter (OTC) family (29). Particularly, genetic variation in the OTC1 is well correlated to the therapeutic efficacy of metformin treatment (29). In this work, we observed an accumulation of metformin in both CCA cells, implying that metformin was transported into the cells and exhibited its effects.

Metformin treatment greatly inhibits the biosynthesis of acetyl CoA which is partly generated by pyruvate dehydrogenase complex (PDC) (30). Moreover, it has been demonstrated that metformin reduces the activity of mitochondrial complex I (12, 31) and causes alterations in the ETC. As a result, NADH-contained electrons are not effectively transported through the ETC, thus decreasing the NAD^+^ level (16, 32). Consistent with these studies, we detected increased glucose and fumarate, but decreased NAD^+^ in metformin-treated HUVEC cells. It seems that the accumulation of TCA cycle intermediates may facilitate the biosynthesis of alanine and aspartate for normal epithelial cells.

On the contrary, we observed raised levels of NAD^+^ and declined levels of TCA cycle intermediates in metformin-treated CCA cells. This discrepancy might result from the metabolic transformation of cancer cells. Cancer cells produce energy through the aerobic glycolysis, in which high rates of glycolysis and lactic acid fermentation occur in the cytosol regardless of the oxygen level (33, 34). Given the enhanced glucose consumption and lactate production, we assume that metformin treatment boosts glycolysis and drains the electrons from NADH for the conversion of pyruvate to lactate, thus aggravating the Warburg effect in cancer cells. It is well known that the regulation of glycolysis is highly associated with AMPK, which can be activated by metformin (19, 35, 36). Similarly, our results indicate that glycolysis is profoundly affected by metformin in CCA cells, providing independent support for the viewpoint that AMPK is vital for the anticancer effect of metformin treatment on CCA cells.

Biosynthesis of amino acids is tightly linked to the TCA cycle and glutamate metabolism. Our results show that metformin treatment decreases glutamate, glutamine, asparagine and β-alanine in both CCA cells and HUVECs, indicating that metformin has a significant effect on the synthesis of non-essential amino acids. The lack of substrates in metformin-treated cells potentially leads to insufficient glutathione, thus interrupting the oxidative stress.

We found that the levels of three BCAAs were basically unchanged in HUVEC cells but significantly enhanced in metformin-treated CCA cells. As essential amino acids, BCAAs cannot be synthesized endogenously, and they are catabolized by highly reversible enzymes using all three BCAAs as substrates. Therefore, levels of the three BCAAs display the same changing treads with similar variation amplitudes (37). Moreover, metformin suppresses expressions or activities of BCAAs catabolic enzymes (38, 39). Accordingly, we hypothesize that the accumulation of BCAAs in CCA cells might be due to autophagy-induced degradations of proteins. Previous works have well demonstrated that metformin can significantly contribute to the inhibition of mTOR (20, 21), directly leading to the activation of autophagy (40, 41). Consistently with these works, our results provide independent support for the viewpoint that metformin treatment may exert anticancer effect through activating the process of autophagy. It has been showed that BCAAs (particularly leucine) are potent activators of mTORC1 (42), implying the presence of negative feedback regulation from metformin to mTOR signaling. Future research is needed to better understand the correlation between metformin and BCAAs in CCA.

It was previously reported that metformin treatment can induce cell cycle arrest in several other cancer cell lines (43–45). Interestingly, we observed significantly increased levels of UDP-GlcNAc in metformin-treated CCA cells. To our knowledge, this observation represents the first report that the anticancer effect of metformin is related to UDP-GlcNAc. As is known, UDP-GlcNAc is a common donor substrate for the N-glycosylation of most cell-surface receptors and transporters in eukaryotes (46). Previous works indicate that glycoproteins with few N-glycans are significantly up-regulated in a switch-like response to the increased UDP-GlcNAc level, such as TβR, CTLA-4, and GLUT4 which mediate organogenesis, differentiation and cell cycle arrest (47, 48). Contrarily, glycoproteins with high numbers of N-glycans are slowly up-regulated with the increased UDP-GlcNAc level, including EGFR, IGFR, FGFR, and PDGFR which stimulate growth and proliferation of cells (47, 48). Thus, the increased levels of UDP-GlcNAc in the CCA cells could potentially induce enhanced surface levels of low-n glycoproteins, and eventually drive to arrest programs and suppress proliferation. Further studies should be conducted in future to mechanistically understand the relevance between metformin and cell cycle arrest.

Additionally, in order to identify as many metabolites as possible, we chose to record solution NMR spectra on aqueous extracts derived from the cells. Notably, other powerful NMR techniques are able to provide unique advantages in metabolomic analyses. In particular, the high resolution magic angle spinning (HRMAS) NMR spectroscopy offers a window for the observation of individual metabolites in intact tissues (49), which may facilitate the elucidation of molecular mechanisms underlying the anticancer effect of metformin on CCA *in vivo*.

## Conclusions

In this work, we have performed comprehensive NMR-based metabolomic analyses to access the anticancer effects of metformin treatment on two CCA cell lines, and address the underlying molecular mechanisms. We identified characteristic metabolites and significantly altered metabolic pathways for metformin-treated CCA cells relative to untreated controls. Through comparing metformin-induced changes of metabolite levels between the CCA cells and normal HUVEC cells, we suggest that metformin profoundly promotes glycolysis and aggravate the Warburg effect in CAA cells. Moreover, metformin specifically increases BCAAs and UDP-GlcNAc, implying the occurrence of autophagy and cell cycle arrest in metformin-treated CAA cells. These results extend our understanding on the molecular mechanisms underlying the anticancer effect of metformin treatment on CCA cells, and shed light on the clinical use of metformin for CCA managements.

## Data Availability Statement

The original contributions presented in the study are included in the article/**Supplementary Materials**. Further inquiries can be directed to the corresponding authors.

## Authors Contributions

JZ conducted cell biology and NMR experiments and analyzed metabonomic data. CH, TJ, SY, and WS performed NMR analysis and cell culturing. WL was the initiator of this study. DL and JZ wrote the manuscript. All authors contributed to the article and approved the submitted version.

## Funding

This work is supported by The National Science and Technology Major Project of China (No. 2017ZX10203206), the National Natural Science Foundation of China (No. 31971357), Scientific Research Foundation for Advanced Talents, Xiang’ an Hospital of Xiamen University (No. PM20180917008), and the Xiamen Ocean Economic Innovation and Development Demonstration Project (No.16PZP001SF16).

## Conflict of Interest

The authors declare that the research was conducted in the absence of any commercial or financial relationships that could be construed as a potential conflict of interest.

## Supplementary Material

Supplementary Figure 1 2D ^1^H-^1^H TOCSY and ^1^H-^13^C HSQC spectra of aqueous metabolites derived from the Mz-ChA-1 cells. **(A)** ^1^H-^1^H TOCSY spectra; **(B–D)** ^1^H-^13^C HSQC spectra. Resonance assignments are labeled in the spectra.

Supplementary Figure 2 Multivariate statistical analyses of NMR spectral data derived from aqueous extracts of the Met and Ctrl groups of both CCA cells. **(A, B)** PLS-DA scores plots of the Met-Mz group vs. the Ctrl-Mz group **(A)**, and the Met-939 group vs. the Ctrl-939 group **(B)**; **(C, D)** PLS-DA cross-validation plots of Met-Mz vs. Ctrl-Mz **(C),** and Met-939 vs. Ctrl-939 **(D)**; **(E, F)** Hierarchical cluster plots of Met-Mz vs. Ctrl-Mz **(E)**, and Met-939 vs. Ctrl-939 **(F)**.
