# Supplementary figures and images for: Nuclear Magnetic Resonance-Based Metabolomic Analysis of the Anticancer Effect of Metformin Treatment on Cholangiocarcinoma Cells
Source: Front Oncol. 2020 Nov 30;10:570516. doi: 10.3389/fonc.2020.570516 (PMC7735195; doi:10.3389/fonc.2020.570516)

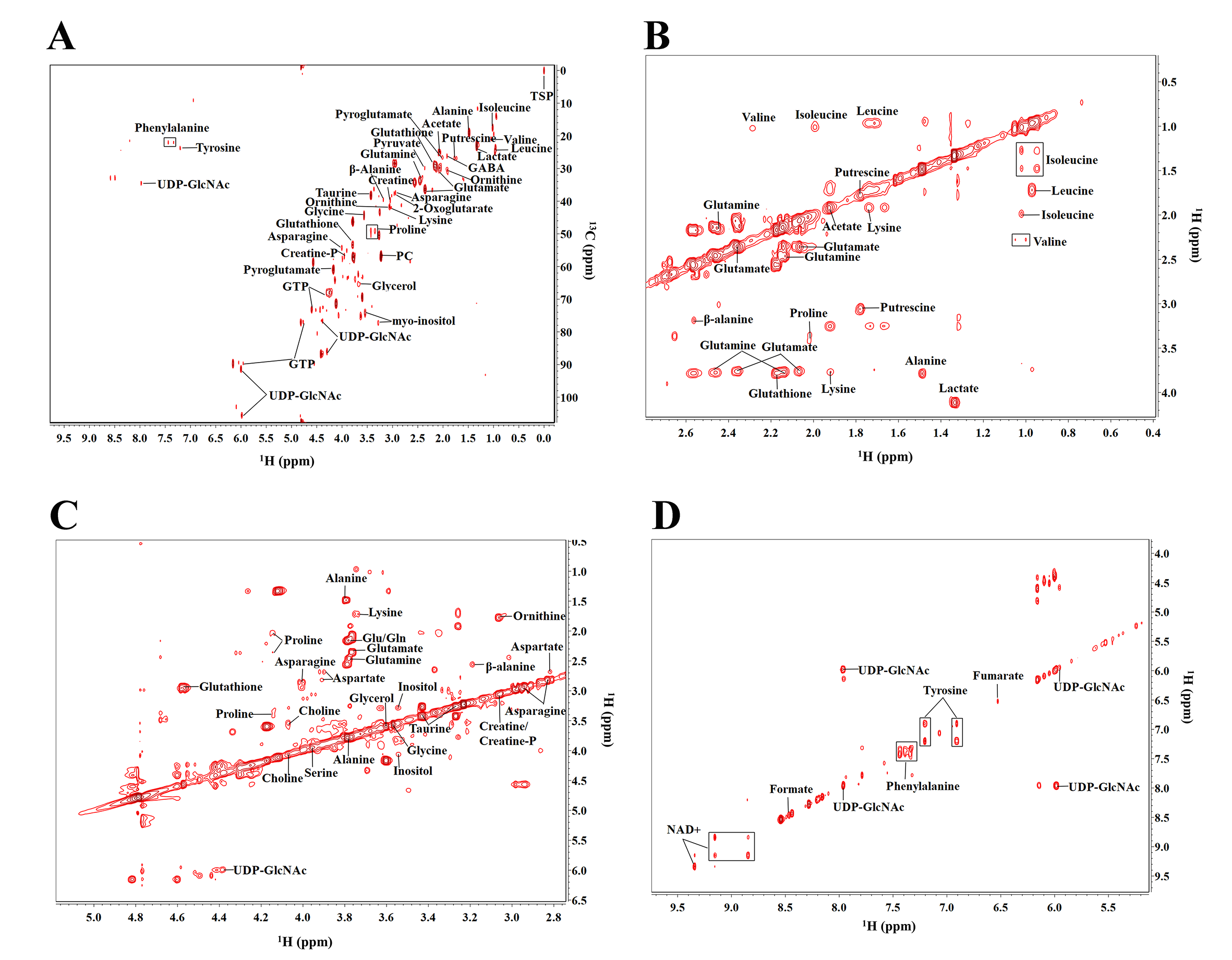

Supplement: Supplementary Figure 1 — 2D 1H-1H TOCSY and 1H-13C HSQC spectra of aqueous metabolites derived from the Mz-ChA-1 cells. (A) 1H-1H TOCSY spectra; (B–D) 1H-13C HSQC spectra. Resonance assignments are labeled in the spectra. [file Image_1.tif]

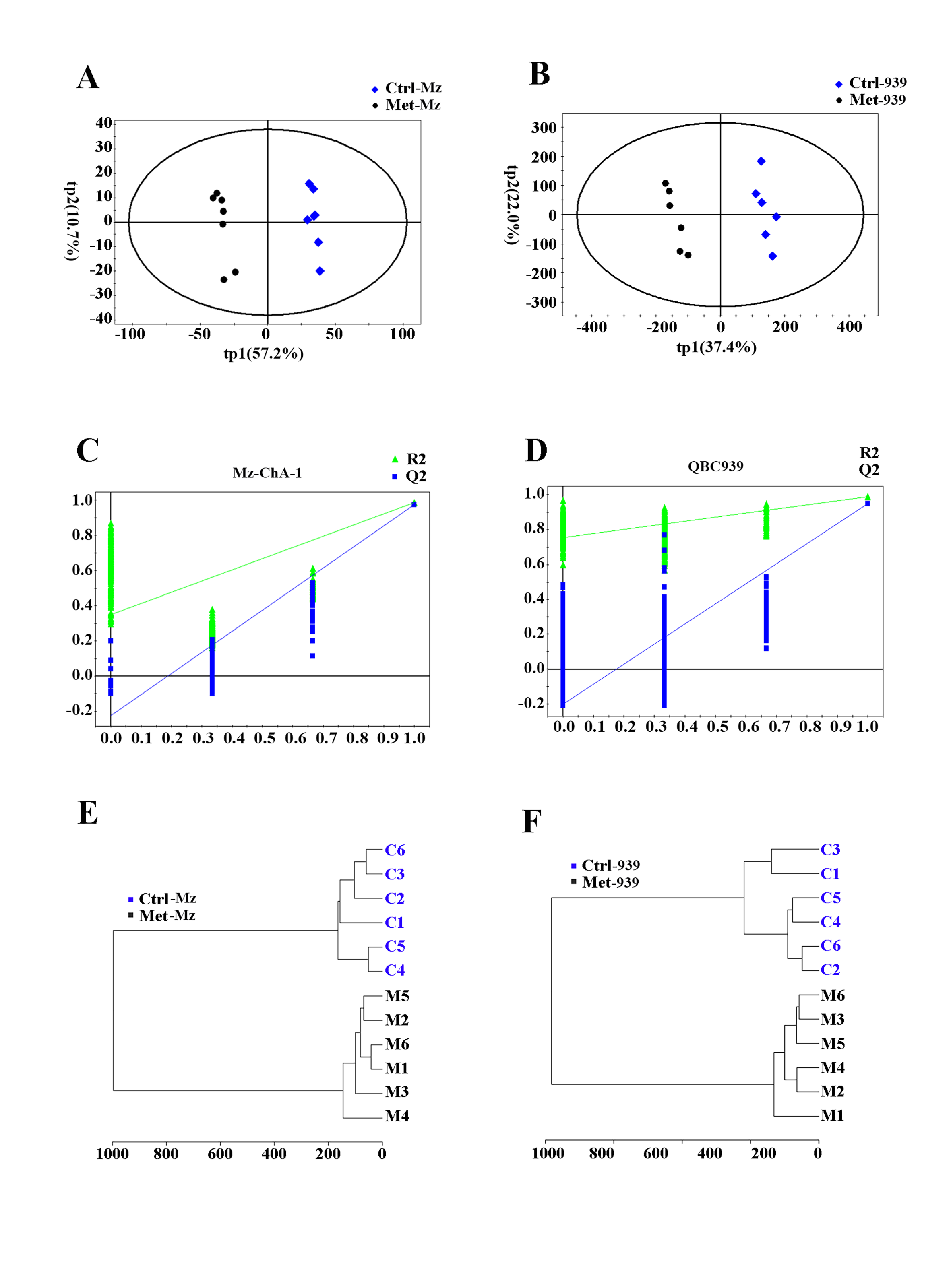

Supplement: Supplementary Figure 2 — Multivariate statistical analyses of NMR spectral data derived from aqueous extracts of the Met and Ctrl groups of both CCA cells. (A, B) PLS-DA scores plots of the Met-Mz group vs. the Ctrl-Mz group (A), and the Met-939 group vs. the Ctrl-939 group (B); (C, D) PLS-DA cross-validation plots of Met-Mz vs. Ctrl-Mz (C), and Met-939 vs. Ctrl-939 (D); (E, F) Hierarchical cluster plots of Met-Mz vs. Ctrl-Mz (E), and Met-939 vs. Ctrl-939 (F). [file Image_2.tif]
